# Unveiling the interplay between rational, psychological and functional factors in continuous glucose monitoring early adoption: Novel evidence from the Dexcom ONE case in Italy

**DOI:** 10.1186/s12913-024-11195-6

**Published:** 2024-06-18

**Authors:** Francesca Zoccarato, Martina Manzoni, Davide Minotti, Emanuele Lettieri, Andrea Boaretto

**Affiliations:** 1 Department of Management, Economics and Industrial Engineering, Politecnico di Milano, Via Lambruschini 4/B, Milan, 20156 Italy; 2 Personalive srl, Via Durando 38, Milan, 20158 Italy

**Keywords:** Diffusion, Healthcare, Continuous glucose monitoring, Adoption, TAM

## Abstract

**Background:**

The escalating prevalence of diabetes, with its multifaceted complications, poses a pressing challenge for healthcare systems globally. In response, the advent of continuous glucose monitoring (CGM) systems, offering technological solutions for daily diabetes management, presents significant opportunities. However, the widespread adoption faces several barriers, linked both to the technological configuration of the devices and to the psychological dimension of patients. Therefore, this study aims to apply and test a theoretical model that investigates the antecedents of the intention to use Continuous Glucose Monitoring systems.

**Methods:**

The research model was built to unveil the impacts of psychological factors, functional components and rational constructs derived from the Technology Acceptance Model (TAM) on CGM systems sustained adoption. To ensure the comparability of results, we have collected data from people who had used Dexcom ONE Dexcom (San Diego, CA) for the first time for at least one month. Employing Structural Equation Modelling (SEM) techniques, the hypothesized relationships among constructs were assessed.

**Results:**

The analyses confirmed the positive correlation of rational factors to the Intention to Use. Subjective Norm, intended as the physicians’ influence, is positively correlated with the Perceived Usefulness. Trend Arrows, albeit being negatively correlated with Perceived Usefulness, have a positive correlation on Perceived Ease Of Use, reinforcing its mediating effect towards Perceived Usefulness. Among psychological factors, Trust in the CGM technology positively correlates with Intention to Use. Health Literacy is negatively correlated to the Intention to Use.

**Conclusions:**

These findings contribute to theoretical and managerial understanding, providing recommendations to enhance the adoption of CGM systems like Dexcom ONE.

**Supplementary Information:**

The online version contains supplementary material available at 10.1186/s12913-024-11195-6.

## Background

Diabetes is a complex and widespread chronic disease, affecting 422 million people globally and leading to 1.5 million annual deaths [1]. In Italy, 4.5 million people report having diabetes, with an additional 1.5 million undiagnosed cases [2]. In Europe, diabetes-related expenditures reach 143 billion euros annually [3], with 20 billion euros spent in Italy alone. Indirect costs are particularly significant as they are often borne by patients [4].

The daily management of diabetes and the self-monitoring of blood glucose levels present urgent yet complex challenges, as patients’ engagement in these activities cannot be taken for granted [5, 6]. Traditionally, individuals have relied on intermittent fingerstick tests, known as self-monitoring of blood glucose (SMBG).

However, continuous glucose monitoring (CGM) systems have emerged as a revolutionary breakthrough in diabetes technology. CGM systems measure interstitial glucose through a wearable sensor and transmit the data to a receiver or smartphone app, where they are displayed and visualized. CGM devices come in two types, namely intermittently scanned (is) CGMs, requiring periodical scanning of the sensor with the receiver, and real-time (rt) CGMs, providing automatic data transmission at some given intervals of time.

CGM devices offer significant benefits to individuals with diabetes, enhancing clinical and psychological outcomes and empowering daily diabetes management [7, 8]. From a clinical standpoint, patients who regularly use a CGM system experience a significant reduction in dizziness and hypoglycemia, leading to less fatigue and improved sleep quality [7]. Moreover, for patients with type 1 diabetes and insulin-treated type 2 diabetes, CGM usage favors better glycemic control and reduced occurrence of hypoglycemic events [9]. Accordingly, this daily glycemic control translates into better health outcomes, such as increased time spent in the optimal glucose range [10] and a significant reduction in glycated hemoglobin compared to patients using SMBG [11].

From a psychological standpoint, CGM technology reduces the fear of hypoglycemia, as patients can check their glucose levels whenever necessary [12]. This, in turn, has a positive impact on the caregivers, as literature has found that parental distress and familial anxiety are significantly reduced with regular CGM use [13]. Additionally, adopting a CGM system brings substantial benefits to patients’ daily lives. Indeed, CGM devices allow patients to avoid the stressful, painful, and time-consuming process of fingerstick tests [7, 14]. Furthermore, the CGM sensor is perceived by most users as comfortable and unobtrusive [15, 16]. CGM usage is linked to higher patient satisfaction, derived either from the system or the prescribed medical therapy [15], and the perception of a better daily routine, less characterized by diabetes control and more devoted to everyday life activities.

Beyond the patients’ perspective, extant literature suggests that CGM is beneficial also for healthcare professionals. The widespread adoption of CGM system may lead to lower overall healthcare expenditures [10, 12, 17], and it can increase Quality-Adjusted Life Years, being highly cost-effective compared with SMBG and FGM [12]. Indeed, CGM implies a significant reduction in direct costs, as patients can achieve better glycemic control with fewer hypoglycemic events and consequently use less healthcare resources, such as emergency rooms, hospitalizations, or visits [10, 18]. On the other hand, continuous CGM utilization is linked with decreased indirect costs concerning absenteeism and travel expenditures for patients and caregivers too [10].

Nevertheless, both physical and psychological barriers might prevent long-term adherence to CGM devices. Physical barriers include issues with sensor adhesiveness and skin problems at insertion sites, while the economic burden varies between countries and regions [10]. Psychological barriers involve mistrust, information overload, and concerns about body image, which is more pronounced in adolescents and young adults [19, 20].

Furthermore, insufficient training that patients receive about CGM usage and how to interpret the data provided could prevent long-term adherence while patients’ involvement in training programs leads to higher therapy efficiency and satisfaction. Moreover, training on CGM use allows patients to improve their proficiency with the CGM device and their ability to read glucose data, allowing them to take suitable and aware corrective actions [21]. This is in line with the role of the attitude of healthcare professionals, particularly diabetologists, towards CGM systems, as diabetes specialists play a pivotal role in influencing patients’ CGM adoption. Diabetologists’ readiness to advocate for CGM technology has been deeply studied by [22, 23], where the results state that only a minor part (20%) of the healthcare professionals involved show a high degree of readiness and a positive attitude towards CGM technology, therefore they regularly recommend its use to their patient while 39% is classified as not ready to promote CGM technology, as the respondents reported a negative perception of CGM systems. The reluctance of this cluster is mainly due to healthcare professionals’ time constraints and limited expertise in keeping up with technological advancements [19].

Henceforth, CGM constitutes a transformative paradigm shift poised to revolutionize the management of diabetes. However, its innovative nature introduces a plethora of acceptance challenges, encompassing physical, psychological, and the level of support from healthcare professionals, which may impede patients’ adoption rates. Acceptance stands as a pivotal determinant in forecasting the diffusion trajectory of health digital technologies over time [24], playing a fundamental role in gauging their potential positive impact on the health of diabetic patients.

More specifically, this study endeavors to illuminate the critical factors underpinning CGM adoption, exploring the intricate interplay between rational and psychological components alongside the functional attributes inherent in the device. Notably, the functional attributes will be elucidated through the real-time CGM system Dexcom ONE, serving as the focal point of inquiry in the present investigation. Indeed, by choosing just one system, the assessments of the device characteristics were comparable.

The paper is structured as follows. In the next sub-section, i.e., Research Model, the literature on adoption of new technology is reviewed to highlight the gaps and develop a research framework comprising elements of diverse theories to support the subsequent empirical analysis. In the Methods section, the methodology for data collection and analysis is presented. Results section outlines the findings of the empirical investigation and discusses the major achievements of the paper. Finally, the last section offers a final discussion about the value of the main results for researchers and managers.

## Research Model

Information technology adoption and use have been extensively investigated in extant literature, and significant theoretical and empirical evidence has built a strong case for the Technology Acceptance Model (TAM) [25, 26], also in the specific case of the healthcare field [27–31].

This theory represents one of the most influential refinements and improvements of the Theory of Reasoned Actions (TRA) [32] and the Theory of Planned Behavior (TPB) [33] in evaluating the acceptance of information system technology and IT use behavior. According to the model, the Actual System Use (i.e., the endpoint where people use the technology) is determined by the Behavioral Intention to Use (ITU), a factor that leads people to use the technology. The more positive the intention toward using a technological system, the higher will be the usage of such a technology. The framework suggests that there are two primary factors positively influencing the intention toward using a specific technology: Perceived Usefulness and Perceived Ease of Use. Perceived Usefulness (PU) was defined by Davis 1989 as “the degree to which a person believes that using a particular system would enhance their job performance” [25]. On the other hand, Perceived Ease of Use (PEOU) refers to “the degree to which a person believes that using a particular system would be free from effort” [26]. Also, the more a technology is perceived easy to use, the more it is perceived as useful. From this theory, we retrieved the first three hypothesis of our model.

### H1

*Perceived Usefulness has a positive impact on the Intention to Use the CGM system.*

### H2

*Perceived Ease of Use has a positive impact on the Intention to Use the CGM system.*

### H3

*Perceived Ease of Use has a positive impact on Perceived Usefulness of the CGM system.*

The original framework of the TAM has undergone subsequent extensions aimed at further exploring the causal antecedents governing the Perceived Usefulness (PU) and Perceived Ease of Use (PEOU) constructs. Particularly noteworthy is TAM2, which integrates the influence of social processes through the inclusion of Subjective Norm. This construct assesses “a person’s perception that most people who are important to him/her think he should or should not perform the behavior in question” [34]. Unlike other extensions such as UTAUT [35], TAM2 has shown how Subjective Norm exerts a positive impact on Perceived Usefulness.

In the specific case, physicians’ opinions and suggestions have been identified as crucial factor in patients’ adoption of CGM [36, 37]. With the appropriate guidance from physicians, patients can develop a comprehensive understanding of the effectiveness and benefit of such a device in their health management [38]. For this reason, we have operationalized the Subjective Norm into the physicians’ opinions and investigated their role as an antecedent of the Perceived Usefulness toward the Intention to Use.

### H4

*Subjective Norm has a positive impact on Perceived Usefulness of the CGM system.*

TAM posits that the influence of external variables such as the intention to use is mediated by Perceived Usefulness and Perceived Ease of Use [39]. Technological characteristics of health devices have been empirically demonstrated to correlate with patients’ perceptions, consequently impacting their intention to utilize them [40]. This has also been shown to be a driver or barrier in the case of CGM devices [41].

Therefore, in light of the Dexcom ONE Continuous Glucose Monitoring (CGM) device serving as the reference technology, we have incorporated its principal characteristics, namely the Visibility of Glucose Data, i.e., the core functions of the app that relate to daily glucose monitoring, Trend Arrows, and Alarm functionalities. Trend Arrows show the anticipated glucose trend, which is estimated through the analysis of previous glycemic data, while Alarms are triggered as soon as the glycemic level reaches the hyperglycemic (upper) or the hypoglycemic (lower) threshold. We included these three characteristics because they emerged as the most relevant ones from previous interviews with patients.

### H5

*Visibility of Glucose Data has a positive impact on Perceived Usefulness of the CGM system.*

### H6

*Visibility of Glucose Data has a positive impact on Perceived Ease of Use of the CGM system.*

### H7

*Trend Arrows have a positive impact on Perceived Usefulness of the CGM system.*

### H8

*Trend Arrows have a positive impact on Perceived Ease of Use of the CGM system.*

### H9

*Alarms have a positive impact on Perceived Usefulness of the CGM system.*

### H10

*Alarms have a positive impact on Perceived Ease of Use of the CGM system.*

However, the literature on health devices, particularly CGM systems, has qualitatively identified additional factors that influence adoption, directly affecting the intention to use and deviating from the traditional constructs of TAM2. Consequently, we have incorporated ad-hoc constructs to enhance the explanatory power of our model.

The psychological dimension of stigma has garnered considerable research attention, especially in relation to diabetes, where it correlates with increased symptoms of depression and anxiety [42]. CGM usage may evoke concerns regarding physical attractiveness, body image, and draw people’s attention to the presence of the device [7, 20, 43]. Stigma thus arises when individuals perceive themselves as diverging from the broader social collective, potentially leading to negative impacts on self-esteem and social identity [44]. The stigma associated with medical conditions is widely recognized as a significant barrier to patient engagement in care [45]. However, qualitative insights indicate this is not the result of the rational assessment of the Perceived Usefulness and Perceived Ease of Use, but rather it directly influences the patient’s behavior.

### H11

*Stigma has a negative impact on Intention to Use the CGM system.*

Discrepancies in CGM measures might lead to a lack of trust in the system’s accuracy among patients. Specifically, there is a significant correlation between patients’ trust in the accuracy and reliability of data and their continued usage of the CGM system [19].

The model presented, therefore, includes trust in technology reliability, defined as the extent to which patients perceive the technology (i.e., CGM systems) as dependable and the level of confidence they experience while utilizing it.

### H12

*Trust has a positive impact on Intention To Use the CGM system.*

On the basis of the extant literature and taking into consideration the context of the research, five control variables were added to the model, namely Gender (GEN) [35], Age [46], the presence of caregivers (CAR) [47], the type of diabetes, and Health Literacy (HL) [48].

**Fig. 1:**
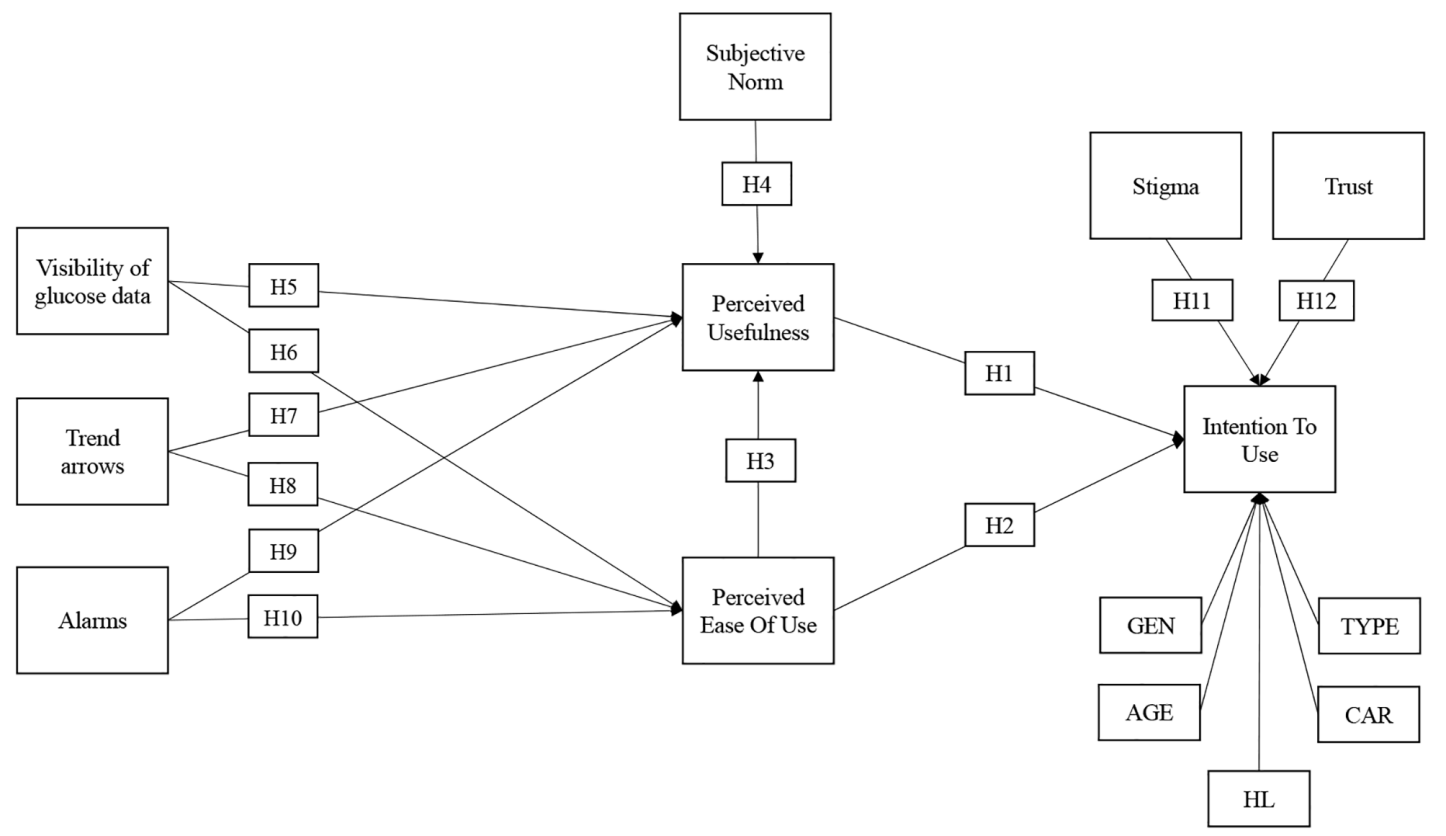
Research model

The research model, together with the control variables, is shown in Fig. 1.

## Methods

### Measurement Development and Data Collection

The proposed model was tested through a quantitative methodology. A survey method was used to collect data. The questionnaire is designed to collect data for the validation of the model in Fig. 1.

The first part is dedicated to gathering general information about the respondents, encompassing personal and demographic details, as well as an exploration of their diabetes-related status, familiarity with digital tools, and the tool they were using for glucose monitoring prior to Dexcom One.

To this end, multiple items were included in the questionnaire, each derived from existing literature for each construct. Specifically, items were retrieved from well-validated scales in the TAM literature for the constructs of Intention to Use [25, 35], and Subjective Norm [39, 49]. Validated scales were also used for the constructs of Stigma [50], Trust [51], and Health Literacy [52]. Each construct relied on three or four validated items, thus partially avoiding the risks and problems associated with scale development [53]. All the items have been measured through a 5-points Likert scale. The functionalities were measured through the frequencies patients had used each of them. The items are shown in Table 1 and a sample questionnaire can be found in the supplementary material.

**Table 1:** Constructs, measurement items and relevant measurement properties of the proposed model

CONSTRUCT	ITEM	MEASUREMENT ITEM	FACTOR LOADING	CR	AVE
Intention To Use (ITU)	ITU1	I intend to use this CGM system in the future.	0,972	0,983	0,952
	ITU2	I predict I would use this CGM system in the next months.	0,983		
	ITU3	I plan to use this CGM system in the next months.	0,972		
Perceived Usefulness (PU)	PU1	I find this CGM system useful in my daily life.	0,834	0,901	0,753
	PU2	Using this CGM system would enable me to manage my health more effectively.	0,871		
	PU3	I would find this CGM system useful to manage my diabetes.	0,897		
Perceived Ease Of Use (PEOU)	PEOU1	Learning how to use the CGM sensor and the relative application is easy for me.	0,865	0,938	0,834
	PEOU2	Interacting with the CGM does not require a big mental effort.	0,898		
	PEOU3	My interaction with the CGM system is clear and understandable.	0,974		
Stigma	STIGMA1	To avoid negative reactions, I don’t tell people I have a CGM system.	0,843	0,873	0,698
	STIGMA2	I had negative consequences at work and/or in my personal relationships because of my CGM system.	0,731		
	STIGMA3	I feel embarrassed when I have to show my CGM system in public.	0,922		
Trust	TRUST1	I think this CGM system is very reliable.	0,919	0,930	0,817
	TRUST2	This CGM system functions the same way each time I use it.	0,811		
	TRUST3	I can fully rely on this CGM system while managing diabetes.	0,974		
Subjective Norm (SN)	SN1	My doctor thinks I will continue to use this CGM system in the future.	0,979	0,98	0,941
	SN2	My doctor thinks it would be a good idea to continue using this CGM system in the future.	0,983		
	SN3	My doctor expects of me to continue using this CGM system in the future.	0,949		
Health Literacy	HL1	I am able to access information about diabetes and CGM technologies.	0,715	0,916	0,736
	HL2	I am able to understand the information about diabetes and CGM technologies.	0,948		
	HL3	I am able to evaluate and judge the information about diabetes and CGM technologies.	0,975		
	HL4	I am able to use the information on diabetes and CGM technologies to make decisions about my health.	0,763		

The questionnaire was created with the aid of the digital platform SurveyMonkey and was designed to be completed via Computer-Assisted Web Interviewing (CAWI). The survey was validated through 20 preliminary interviews conducted by phone, and minor adjustments were made to the questionnaire to ensure its comprehensibility.

The survey was administered to a sample of patients enrolled in 45 hospitals in the south of Italy. These diabetic patients were identified by their doctors, who were asked to select patients eligible to be treated with Dexcom ONE, as patients for such a device had to be insulin-treated. The period for identifying and involving patients, as well as collecting data, was from July 1 to December 31, 2023. The selected sample is particularly relevant as these patients were early adopters, being the first one in Italy to adopt the device.

Selected patients were provided with the device to test it, and if they were still using the device after one month, they were asked to answer to the questionnaire. In this way, 360 patients were identified as eligible to be included in the sample and were asked to participate in the study. To ensure homogeneity of responses, after one month, they were contacted to complete the questionnaire. A total of 235 patients returned the questionnaires (65%). All collected data were checked for consistency to minimize data entry errors and partially completed questionnaires. As a result, 157 valid responses were included (33% dropout rate). Indeed, we did not analyze partially completed questionnaires. Furthermore, data collection was performed in compliance with GDPR regulations, with respondents being informed and assured of anonymity.

### Data analysis

Once collected, the data were analyzed. The initial step involved a descriptive examination of personal, demographic, and health-related questions. Subsequently, data analysis was conducted using the partial least squares (PLS) method, a structural equation modeling technique, via the software STATA 17, consistent with previous research [28, 29]. First of all, a Kaiser-Meyer-Olkin (KMO) test was employed to determine the suitability of the sample for factor analysis [54]. The KMO score was above 0.7, indicating that the sample is adequate for conducting an Exploratory Factor Analysis (EFA). Following this, an EFA was conducted using the Principal Component Methodology, establishing the psychometric validity of the scales through construct reliability with Cronbach’s alpha [55]. The validity and consistency of the measurement method for the constructs were evaluated through Confirmatory Factor Analysis (CFA) [53], with convergence validity assessed using two indicators: Composite Reliability (CR) and Average Variance Extracted (AVE).

The model was further tested through Structural Equation Modeling (SEM) [56]. Finally, the Goodness of Fit (GOF) was determined using three indicators, including absolute measures such as the Root Mean Square Error of Approximation (RMSEA) and incremental measures like the Comparative Fit Index (CFI) and the Tucker-Lewis Index (TLI).

## Results

Among the 157 respondents, 58% were male and 42% were female. Regarding diabetes type, 42% reported type 1 diabetes while 58% reported type 2 diabetes. Half of the sample received support from a caregiver. The majority of respondents (63%) reported using traditional glucometers, followed by 21% using flash glucose monitoring, 11% using rt-CGM, and 5% not using any monitoring device. Further details regarding the sample can be found in Table 2.

**Table 2:** Socio-Demographic characteristics of the sample

Age		Occupation		Education		Time of Diagnosis	
< 13	0,6%	Worker/employed	34%	Post-Degree/Master/PhD	3%	< 6 months	5%
14–17	1,3%	Student	5%	Degree (5 years)	6%	7 months − 1 year	4%
18–24	3,8%	Unemployed	8%	Degree (3 years)	8%	1–2 years	4%
25–34	5,7%	Retired	41%	High school Diploma	39%	3–5 years	9%
35–44	12,1%	Houseman/Housewife	11%	Primary/middle school diploma	44%	> 5 years	78%
45–54	12,7%						
55–64	22,3%						
> 65	41,5%						

Additionally, it is noteworthy that respondents, on average, scored 3.66 out of 5 on a Likert scale measuring health literacy. This indicates a moderate level of health literacy among the respondents, suggesting an awareness of their condition and a certain degree of ability to comprehend and utilize diabetes-related information for managing their daily lives and health.

Both EFA and CFA validated the association between items and latent variables as shown in Table 1.

Subsequent to this, the structural equation model (SEM) affirmed the suitability of the model. Specifically, it confirmed the validity of both the relationship between Perceived Usefulness (PU) and Intention to Use (ITU), and Perceived Ease of Use (PEOU) and ITU. Furthermore, PEOU was observed to correlate with ITU, mediated by PU. Social Norm (SN) was found to be correlated with PU. ITU exhibited correlation with Trust (TST) but not with Stigma (STG). Notably, the only feature that displayed correlation with both PU and PEOU was the utilization of Trend Arrows, albeit with divergent effects: a negative correlation with PU and a positive correlation with PEOU. The mediating effect of PEOU in the relationship between Trend Arrows and PU was verified through a Sobel-Goodman test (*p*-value: 0.005). Other characteristics of the device did not exhibit correlation with either PU or PEOU. Table 3 presents the outcomes of hypotheses testing, encompassing standardized coefficients, standard errors, and *p*-values.

**Table 3:** Hypothesis testing results

Hypothesis	Path	Coef.	Std. Err.	*p*-value	Results
H1	PU ◊ITU	0.861	0,0,42	0.000***	**Significant**
H2	PEOU ◊ITU	-0.145	0.072	0.045**	**Significant**
H3	PEOU ◊PU	0.332	0.082	0.000***	**Significant**
H4	SN ◊PU	0.723	0.053	0.000***	**Significant**
H5	VAL ◊PU	0.010	0.071	0.884	Not Significant
H6	VAL ◊PEOU	-0.071	0.089	0.428	Not Significant
H7	ARR ◊PU	-0.175	0.078	0.026**	**Significant**
H8	ARR ◊PEOU	0.561	0.079	0.000***	**Significant**
H9	ALL ◊PU	-0.070	0.057	0.224	Not Significant
H10	ALL ◊PEOU	-0.003	0.074	0.973	Not Significant
H11	STG ◊ITU	-0.041	0.049	0.399	Not Significant
H12	TST ◊ITU	0.144	0.085	0.091*	**Significant**

* *p*-value < 0.1 **p-value < 0.05 ***p-value < 0.001

In Table 4, the correlation analysis revealed that among the control variables, only Health Literacy (HL) exhibited a significant correlation with Intention To Use (ITU). This suggests that higher levels of health literacy are associated with greater intention to utilize the CGM system among diabetes patients.

**Table 4:** Incidence of control variables on ITU

Item	Control Variable	Coef.	Std.Err.	*p*-value	Results
HL	Health Literacy	-0.092	0.054	0.087*	**Significant**
GEN	Gender	0.040	0.046	0.381	Not Significant
AGE	Age	-0.086	0.055	0.118	Not Significant
TYPE	Type of Diabetes	-0.033	0.052	0.520	Not Significant
CAR	Presence of Caregiver	0.055	0.047	0.246	Not Significant

* *p*-value < 0.1 **p-value < 0.05 ***p-value < 0.001

**Fig. 2:**
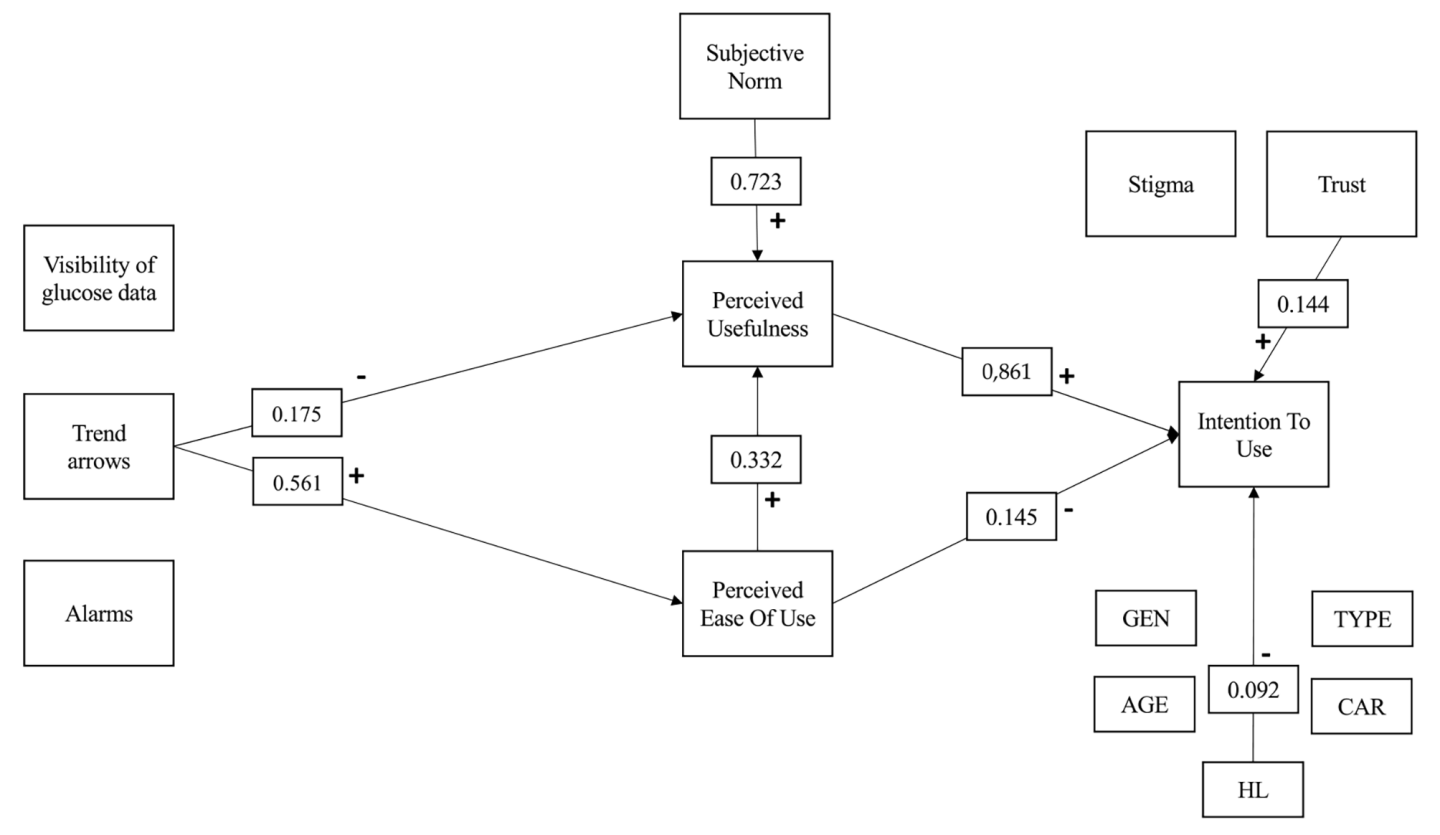
Tested model and the significant relations among the constructs

Figure 2 shows the tested model and the significant relations among the constructs.

Two of the four Goodness of Fit indices were deemed fully acceptable, while one was found on the borderline of acceptability. GOF values and thresholds are shown in Table 5.

**Table 5:** Goodness of Fit indicators

Indicator	Threshold	Value
Square error of approximation (RMSEA)	< 0.1	0.097
Comparative fit index (CFI)	> 0.85	0.888
Tucker-lewis index (TLI)	> 0.85	0.875

## Discussion

The contribution of this study is twofold. From a theoretical standpoint, it introduces a novel perspective by elucidating the interaction among rational, psychological, and functional elements in the acceptance and adoption of Continuous Glucose Monitoring (CGM) systems. This innovative approach provides insights into how specific characteristics can promote patients’ intention to use CGM systems. Moreover, the study considers the influence doctors and nurses, alongside psychological factors like Stigma and Trust, to comprehend their impact on intention to use. This theoretical advancement yields practical insights into the effective integration of CGM systems into patients’ daily lives.

All relationships examined within the Technology Acceptance Model (TAM) were found to be statistically significant. Notably, an enhanced perception of Continuous Glucose Monitoring (CGM) systems’ usefulness positively correlates with patients’ willingness to utilize them. Interestingly, perceived ease of use demonstrates a negative correlation with intention to use. However, when mediated through perceived usefulness, it positively correlates with intention to use. This underscores the importance of effectively communicating the benefits of CGM technology to patients, emphasizing its role in enhancing diabetes management and improving clinical outcomes and quality of life. However, a noteworthy finding emerges from the negative correlation between perceived ease of use and intention to use. Similarly, health literacy and intention to use CGM systems are negatively correlated. This suggests that patients with higher health literacy, possessing a deeper understanding of their condition, may have higher expectations of CGM devices or may be cautious about adopting technology due to perceived complexity and increased responsibility. Those transitioning from SMBG may express concerns about accuracy discrepancies between instruments. This highlights the need for personalized CGM therapy guided by healthcare professionals, who can assist health-literate individuals in actively utilizing advanced CGM devices and empower less health-literate individuals to maximize the benefits of basic systems.

The study underscores the pivotal role of physicians, especially diabetologists, in promoting CGM adoption. Tailored recommendations from healthcare professionals, considering the diverse profiles of patients, are crucial to fostering the adoption of CGM systems.

The study places emphasis on user-friendliness and intuitiveness of CGM systems, urging manufacturers to direct their efforts toward enhancing these aspects. Healthcare professionals are encouraged to provide comprehensive education to patients, enabling them to unlock the full potential of CGM technology.

The use of Trend Arrows affects both the perceived ease of use and the perceived usefulness with contrasting effects. If a higher usage frequency of Trend Arrows positively impacts the perceived ease of use, perceived usefulness is negatively correlated with a regular use of the function. This dichotomy appears to stem from users’ excessive reliance on Trend Arrows, which may consequently result in the inadvertent neglect of other pivotal facets of glucose monitoring or induce distress related to data interpretation. Consequently, there is a pertinent need for healthcare professionals to educate patients on the appropriate utilization of Trend Arrows and advocate for periodic refreshers to rectify any errors that may arise over time.

Trust, in terms of in the accuracy and reliability of data, is another key aspect examined, revealing its direct positive correlation with the behavioral intention. Addressing trust-related concerns among patients becomes crucial, and healthcare professionals are encouraged to collaborate with manufacturers to find solutions for these psychological barriers.

In conclusion, this study highlights the potential benefits of the CGM technology in diabetes management, while underscoring the various challenges and concerns that must be addressed for its widespread adoption. Moreover, it emphasizes the critical role of all those involved in supporting people with diabetes, such as health care professionals, manufacturers and patient associations in ensuring the effective utilization and acceptance of CGM systems by individuals with diabetes.

### Limitations of the study and further research

The research is subject to several limitations. Firstly, the sample size remains constrained, indicating a necessity for further studies to broaden the sample pool. This expansion could involve including individuals from diverse regions across Italy, as well as participants from othercountries.

Secondly, the recruitment process assessed patient responses after only one month of the Dexcom ONE use, thus lacking insights into the adaptation phase of device usage and potential changes in perceptions over a prolonged duration. Consequently, conducting a longitudinal analysis could provide valuable insights into how perceptions of device functionalities evolve over time. Furthermore, to ensure comprehensive assessment and comparability of results on the same characteristics, data collection was limited to experiences with a single device. However, investigating the performance of various characteristics across different devices could offer further and relevant insights.

Thirdly, it’s important to note that the model focuses on the intention to use the CGM system rather than the intention to continuously use it over time. This distinction is significant because all patients in the study had already used the CGM system for at least one month. Despite this, the model was chosen due to its relevance in capturing the factors influencing initial acceptance and adoption decisions. Thus, future research may consider incorporating additional controls or exploring factors related to sustained usage and device switching behavior.

Lastly, while the current model investigates the influence of three specific device characteristics, including additional attributes would contribute to a more comprehensive understanding of their collective impact on the intention to utilize such device.

## Conclusion

The present study endeavors to explore the determinants influencing the acceptance and subsequent adoption of Continuous Glucose Monitoring (CGM) systems among individuals with diabetes. Specifically, the model illuminates the dynamic interplay between rational and psychological factors, augmented by the introduction of the device characteristics, aimed at bolstering the intention to utilize such systems. To enhance comparability in assessing device characteristics, the study leverages the Dexcom ONE CGM as a representative case.

Overall, the findings offer a significant contribution to established models by elucidating the pivotal role of device characteristics in shaping device acceptance. Additionally, the study yields practical implications and recommendations for fostering the adoption of such devices, with the potential to enhance patients’ quality of life and optimize care delivery.

### Electronic supplementary material

Below is the link to the electronic supplementary material.

Supplementary material: Questionnaire: The file contains the questionnaire submitted to patients, purposefully developed for this study. More in detail, the questionnaire is made by three sections: part 1 (demographics and personal information), part 2 (Model Measurement) and part 3 (Usage of Dexcom ONE functions). Part 2 and part 3 have been measured through Likert Scale from 1: strongly disagree to 5: strongly agree.

## Data Availability

The datasets generated and analyzed during the current study are not publicly available. Although we have removed identifying information, we cannot risk identification by making the data available for public inspection, as we guaranteed anonymity to respondents. Datasets could be available from the corresponding author on reasonable request.

## Abbreviations

CGM: Continuous Glucose Monitoring
is-CGM: intermittently scanned Continuous Glucose Monitoring
rt-CGM: real-time Continuous Glucose Monitoring
SMBG: Self Monitoring of Blood Glucose
ITU: Intention To Use
PU: Perceived Usefulness
PEOU: Perceived Ease of Use
GEN: Gender
CAR: Presence of caregivers
HL: Health Literacy
TAM: Technology Acceptance Model
