# Supplementary material for: Unveiling the interplay between rational, psychological and functional factors in continuous glucose monitoring early adoption: Novel evidence from the Dexcom ONE case in Italy
Source: BMC Health Serv Res. 2024 Jun 18;24:747. doi: 10.1186/s12913-024-11195-6 (PMC11186290; doi:10.1186/s12913-024-11195-6)
Supplement: Supplementary file 1 — Supplementary material: Questionnaire: The file contains the questionnaire submitted to patients, purposefully developed for this study. More in detail, the questionnaire is made by three sections: part 1 (demographics and personal information), part 2 (Model Measurement) and part 3 (Usage of Dexcom ONE functions). Part 2 and part 3 have been measured through Likert Scale from 1: strongly disagree to 5: strongly agree. [file 12913_2024_11195_MOESM1_ESM.pdf]

| # | Questions | Answers |
|---|---|---|
| 1 | You are: | • Person with diabetes<br>• Caregiver of person with diabetes |
| 2 | Gender: | • Female<br>• Men<br>• None of the above<br>• I prefer not to declare it |
| 3 | What is your year of birth? | |
| 4 | What is your most recent educational qualification? | • Postgraduate/Doctorate<br>• Master's degree (5 years)<br>• Bachelor's degree (3 years)<br>• Diploma<br>• Elementary/Middle school |
| 5 | What is your employment situation? | • Worker/worker<br>• Student<br>• Not employed<br>• Retired<br>• Homemaker<br>• Other (specify) |
| 6 | Please indicate your region of residence: | |
| 7 | What type of diabetes do you suffer from? | • Type 1<br>• Type 2<br>• Other (specify) |
| 8 | How long have you been diagnosed with diabetes? | • Less than 6 months<br>• 7 months to 1 year<br>• 1 to 2 years<br>• 3 to 5 years<br>• More than 5 years |
| 9 | In the diabetes treatment you have indicated, is insulin administration required? | • Yes (= more than two injections per day)<br>• Basal insulin only (= one injection per day)<br>• No, treatment involves oral or injectable non-insulin drugs<br>• No, treatment involves a specific diet |
| 12 | Before you were prescribed the Dexcom One continuous glucose monitoring system, what were you using for diabetes management? | • Nothing, I have just been diagnosed with diabetes<br>• Glucometer<br>• Flash Glucose Monitoring device - FGM (data collection is not automatic, but requires periodic scans) |
| 15 | How often did you access the app on your smartphone/receiver linked to your previous intermittent device? | • Two/three times a week<br>• Once/twice a day<br>• Two/three times a day<br>• Five times a day or more |
| 18 | How often did you use to take blood glucose measurements with the glucose meter? | • At least 1 time per month<br>• At least once a week |

| | | |
|---|---|---|
| | | • Once/twice a day<br>• Three/four times a day<br>• Five or more times a day |
| 19 | Think now of the Dexcom One continuous glucose monitoring system. Have you been using it continuously for the last 30 days? | • Yes<br>• No |

**Part 2 – Likert Scale**

| # | Question | 1 - Complete disagreement | 2 - Disagreement | 3 - Indifference | 4 - Agreement | 5 - Complete agreement |
|---|---|---|---|---|---|---|
| 20 | I am able to access information about diabetes and CGM technologies. | | | | | |
| 21 | I am able to understand the information about diabetes and CGM technologies. | | | | | |
| 22 | I am able to evaluate and judge the information about diabetes and CGM technologies. | | | | | |
| 23 | I am able to use the information on diabetes and CGM technologies to make decisions about my health. | | | | | |
| 24 | I find this CGM system useful in my daily life. | | | | | |
| 25 | Using this CGM system would enable me to manage my health more effectively. | | | | | |
| 26 | I would find this CGM system useful to manage my diabetes. | | | | | |
| 27 | Learning how to use the CGM sensor and the relative application is easy for me. | | | | | |
| 28 | Interacting with the CGM does not require a big mental effort. | | | | | |
| 29 | My interaction with the CGM system is clear and understandable. | | | | | |
| 30 | My doctor thinks I will continue to use this CGM system in the future. | | | | | |
| 31 | My doctor thinks it would be a good idea to continue using this CGM system in the future. | | | | | |
| 32 | My doctor expects of me to continue using this CGM system in the future. | | | | | |
| 33 | I intend to use this CGM system in the future. | | | | | |
| 34 | I predict I would use this CGM system in the next months. | | | | | |

| | | | | | | |
|---|---|---|---|---|---|---|
| 35 | I plan to use this CGM system in the next months. | | | | | |
| 36 | To avoid negative reactions, I don’t tell people I have a CGM system. | | | | | |
| 37 | I had negative consequences at work and/or in my personal relationships because of my CGM system. | | | | | |
| 38 | I feel embarrassed when I have to show my CGM system in public. | | | | | |
| 39 | I think this CGM system is very reliable. | | | | | |
| 40 | This CGM system functions the same way each time I use it. | | | | | |
| 41 | I can fully rely on this CGM system while managing diabetes. | | | | | |

**Part 3 – Dexcom ONE functions**

Note: 0 means *“I don’t know the function”*

| # | How often did you use this function in the last month? | I don’t know | 1 - Complete disagreement | 2 - Disagreement | 3 - Indifference | 4 - Agreement | 5 - Complete agreement |
|---|---|---|---|---|---|---|---|
| 42 | Data visibility on smartphone (i.e. actual blood glucose value, blood glucose trend graph, Time In Range) | | | | | | |
| 43 | Visibility of blood glucose trends (i.e. arrow) | | | | | | |
| 44 | Setting and customisation of alarms | | | | | | |
